# Flavonoids and Other Polyphenols Act as Epigenetic Modifiers in Breast Cancer

**DOI:** 10.3390/nu12030761

**Published:** 2020-03-13

**Authors:** Priyanga Selvakumar, Aja Badgeley, Paige Murphy, Hina Anwar, Urvashi Sharma, Katharine Lawrence, Ashakumary Lakshmikuttyamma

**Affiliations:** Department of Pharmaceutical Sciences, Jefferson College of Pharmacy, Thomas Jefferson University, Philadelphia, PA 19107, USA

**Keywords:** flavonoids, Epigenetics, breast cancer, methylation, chromatin modification

## Abstract

Breast cancer is a common cancer that occurs due to different epigenetic alterations and genetic mutations. Various epidemiological studies have demonstrated an inverse correlation between breast cancer incidence and flavonoid intake. The anti-cancer action of flavonoids, a class of polyphenolic compounds that are present in plants, as secondary metabolites has been a major topic of research for many years. Our review analysis demonstrates that flavonoids exhibit anti-cancer activity against breast cancer occurring in different ethnic populations. Breast cancer subtype and menopausal status are the key factors in inducing the flavonoid’s anti-cancer action in breast cancer. The dose is another key factor, with research showing that approximately 10 mg/day of isoflavones is required to inhibit breast cancer occurrence. In addition, flavonoids also influence the epigenetic machinery in breast cancer, with research demonstrating that epigallocatechin, genistein, and resveratrol all inhibited DNA methyltransferase and altered chromatin modification in breast cancer. These flavonoids can induce the expression of different tumor suppressor genes that may contribute to decreasing breast cancer progression and metastasis. Additional studies are required to confirm the contribution of epigenetic modifications by flavonoids to breast cancer prevention.

## 1. Introduction

Breast cancer is one of the most common cancers among women in both developed and developing countries. According to the American Cancer Society, approximately 2 million new cases of breast cancer occurred worldwide in 2018 [1]. Major risk factors for breast cancer include genetic mutation, obesity, alcohol consumption, lack of physical activity, red meat consumption, and processed food intake [2,3,4,5]. However, a variety of factors provide prevention of breast cancer occurrence, including sustaining a healthy weight; consuming a diet rich in produce, whole grains, and beans; and limiting consumption of alcohol, red meat, fatty, salty, high sugar foods, and processed foods [6]. Race and ethnicity are also major factors in the occurrence of breast cancer, with the highest incidence rates among Caucasian and African-American women and lower incidence rates among Asian and Hispanic populations [7]. Compared to Caucasian women, breast cancer is more aggressive in African-American women [8]. This may be related to the significantly higher percentage of promoter methylated genes in African American patients diagnosed with triple negative breast cancer than in Caucasian patients [9]. Nevertheless, Caucasian women, specifically Ashkenazi Jews who are carriers of BRCA1 and BRCA2 mutations, still have higher risk of developing breast cancer [10,11].

In breast cancer, the changes in expression levels of oncogenes and tumor suppressor genes are largely controlled by epigenetic alterations, particularly DNA methylation and histone modifications. Many studies have shown that early intervention of epigenetic alterations can prevent cancer occurrence. Epigenetic alterations often occur in the early stages during cancer development [12,13,14]. Preventing the early alteration of these epigenetic changes may reduce cancer cell proliferation. Hence, epigenetic interferences at an early stage may reduce the severity of cancer growth and metastasis. A myriad of epigenetic modifiers are naturally occurring and readily available as dietary nutrients. Among these dietary nutrients, flavonoids and polyphenols have been identified as a major source of epigenetic modifiers. Studies on the action of flavonoids/polyphenols on epigenetic machinery using an in vivo model system are currently lacking. However, there are reports from studies that have utilized breast cancer cell line models to identify the association between various flavonoids and epigenetic modifications. In this review, we included human case study reports on the effect of flavonoids/polyphenols in reducing breast cancer risk. In regards to the mechanism of action, we compiled results of published studies focusing on the epigenetic modification of flavonoids/polyphenols in cell culture models. The in vitro studies suggest that flavonoids induce the expression of different tumor suppressor genes by epigenetic modifications. Since bioavailability of flavonoids is a major concern, it may not be rational to believe that the results detected in cell culture studies will be reflected in the in vivo system. Additional animal studies and human case studies are required to assess the quantity of a dietary source that is required to induce epigenetic changes.

## 2. Methodology 

The influence of diet in breast cancer prevention has been an active topic of discussion in recent years. To prepare this review, we searched PubMed, USDA, and other scientific data resources for the past 15 years using the following keywords: “Flavonoids,” “Polyphenols,” “Breast cancer,” “Epigenetics,” “Promoter methylation,” and “Histone acetylation.” These keywords were used individually or in different combinations to select those published articles that discussed the effect of flavonoids on epigenetic alterations in breast cancer. The materials discussed on other cancers were not selected. 

### 2.1. Flavonoids

The literature was conclusive in regards to the association between diet pattern and cancer formation as most studies have confirmed the importance of a vegetable- and fruit-enriched diet in reducing cancer development. Fruits and vegetables contain various phytochemicals, such as phenolic acids, flavonoids, carotenoids, stilbenes, and lignans, that have been found to reduce breast cancer progression and metastasis [15,16]. The different types of flavonoids that exist in nature—flavonols, flavonones, flavones, isoflavones, and anthocyanins—are all characterized based on their structure. Most dietary flavonoids other than flavan-3-ols exist in the “glycoside” form. Different β-glucosidases enzymes that are present in the small intestine, including cytosolic β-glucosidase (Cβ-g), lysosomal glucocerebrosidase (GCase), and a brush border bound lactase phloridzine hydrolase (LPH), are involved in the deglycosylation of glycoside form of flavonoids to “aglycone” [17]. The flavonoid aglycones are then subject to different types of conjugations such as methylation, sulfation, and glucuronidation [17,18]. Initially, the flavonoid conjugation process occurs in the small intestine and is then transported to the liver [18]. The absorption and bioavailability vary between different class flavonoids and their sources [19].

Flavonols are abundant in fruits and vegetables, including apples, blueberries, onions, and asparagus. Specifically, quercetin is one of the major flavonols that is present in fruits and vegetables [20]. On the other hand, flavon-3-ols are mostly present in tea, grapes, wine, and cocoa beans. Epigallocatechin-3-gallate (EGCG) is the main flavan-3-ols and is most commonly found in green tea [21]. Apigenin and luteolin are the major flavones and are present in celery, chamomile, thyme, oregano, and parsley [22]. Flavonones are primarily present in citrus fruit peel and Isoflavones, such as genistein and daidzein, are present abundantly in soybeans [23]. Anthocyanidins are present in different berries, cherries, and grapes.

### 2.2. Worldwide Flavonoid Intake

In analyzing the intake of flavonoids worldwide, it is important to consider differences in the availability of foods and in diets around the world. Available reports suggest that the types and amounts of flavonoids used vary between countries. The assessment of polyphenol intake worldwide is available from two resources including the US Department of Agriculture (USDA) and Phenol Explorer [24]. In the USA, Canada, and Europe, the consumption of flavonoids is mainly from tea, coffee, wine, fruits, and vegetables. Whereas, in China, Japan, Korea, and other East Asian countries, isoflavone consumption from soy products and green tea is higher compared to other parts of the world. The daily average intake of isoflavones among Chinese and Japanese populations ranges from 16 to 70 mg/day, whereas consumption in American and European populations is approximately 2 mg/day [25]. 

### 2.3. Flavonoid/Polyphenol Intake and Incidence of Breast Cancer 

The association between flavonoid intake and breast cancer risk in women is inconclusive due to conflicting results from different epidemiological studies. A recent case-control study analyzed 1522 breast cancer cases in Chinese women from 2007 to 2018 and 1547 matched control subjects and demonstrated an inverse correlation between the risk of breast cancer and the consumption of total flavonoids, anthocyanidins, proanthocyanidins, flavanones, flavones, flavonols, and isoflavones [26]. This effect was not related to menopausal or estrogen receptor (ER)/progesterone receptor (PR) status. 

Another case control study identified a dual association between flavonoid intake and breast cancer risk in relation to alcohol consumption [27]. Breast cancer risk was shown to be reduced in women who did not drink alcohol but consumed flavonoids, flavonols, catechins, theaflavins, and proanthocyanidins. Whereas, breast cancer risk was increased in heavy alcohol drinkers.

Another case study carried out in the USA used a total of 1434 breast cancer patients and 1440 control patients [28]. This study identified that the intake of all flavonoids except flavanones, anthocyanidins, and isoflavones reduced the risk of breast cancer in postmenopausal women. However, in premenopausal women, there was no association between breast cancer risk and any form of flavonoid intake. Furthermore, the same research group continued their studies to evaluate for a possible association between flavonoid intake and breast cancer survival. The patient group was diagnosed with invasive breast cancer from 1996–1997 and a survival assessment of 1273 patients was carried out from 2002 to 2004 [29]. The study revealed that total flavonoids, anthocyanidins, and isoflavones were associated with a reduction in breast cancer mortality among postmenopausal women. A moderate reduction in mortality was also observed in premenopausal women with the consumption of flavones and anthocyanidins. A study conducted in Italy using 2569 breast cancer patients and 2588 hospital controls found that flavones intake reduces breast cancer risk, but no correlation was detected for flavanones, flavan-3-ols, anthocyanidins, and isoflavones [30]. These studies indicated that flavonoids exhibit breast cancer preventive properties, however menopausal status is a key determinant in the association between flavonoids and breast cancer prevention. 

A few case studies were focused on only isoflavones and a wide disparity was observed on the outcome of these studies as the results were influenced by type of breast cancer, study region, quantity, and duration of isoflavone intake [31,32,33,34,35,36]. Most of the case control studies that were performed in Asian countries revealed that isoflavone intake reduces the risk of breast cancer, whereas some studies conducted in Western populations observed no correlation. There are also some studies that identified that estrogen-like activity of isoflavones enhances the growth of ER-positive breast tumors [32,33,34]. Recently, a large meta-analysis indicated that the dose of isoflavone is a critical factor for its effect in reducing breast cancer risk. This study showed that a higher amount of daily soy consumption, approximately 10 mg/day, decreased breast cancer risk by 3% (95% CI 1–5%) [37]. Another recent meta-analysis also suggested that a daily consumption of 10mg/day of soy isoflavones reduced breast cancer mortality by 9% [38]. However, no association was identified between soy supplement intake and breast cancer risk. According to supplement manufacturing companies, one tablet of soy supplements can contain 3.75 to 37.50 mg of soy isoflavones. These conflicting reports from various ethnicities and study populations suggest that the dose of the isoflavones and breast cancer subtypes influence the effect of isoflavone in breast cancer prevention. 

Studies have also specifically concentrated on analyzing the correlation between coffee/tea intake and breast cancer incidence. Both tea and coffee contain different groups of polyphenols. One study of Swedish women found that coffee intake significantly decreased the incidence of pre- and post-menopausal breast tumor development in patients, however there was an increased incidence of breast cancer with increasing tea intake [39]. A Canadian research team reported that high coffee consumption (≥5 cups per day) reduced the ER-negative and postmenopausal breast cancer risk [40]. A study conducted in the USA that focused on African-American populations did not observe any association between coffee or tea intake and breast cancer risk [41]. In the Netherlands, a population-based prospective cohort study reported that coffee consumption increased the risk of breast cancer occurrence by two-fold [42]. A meta-analysis identified significant associations between reduction in breast cancer incidence and coffee consumption, while no association was observed among cohort and case-control studies conducted in Europe or the United States. However, no significant association was noted among Asian studies [43]. As was discussed above, green tea consumption is higher in East Asian countries and breast cancer mortality is lower among women from these countries compared to American and European countries. A Japanese research group identified that 10 cups of green tea per day decreased the relative risk of cancer incidence [44]. A recent meta-analysis included 14,058 breast cancer patients and 15,043 control subjects to analyze an association between green tea intake and breast cancer risk [45]. This study found a negative correlation between the risk of breast cancer and green tea intake. Overall, these studies have yielded contradicting results regarding the relationship between coffee and tea consumption and the incidence or risk of breast cancer.

## 3. Epigenetics and Breast Cancer

According to different molecular characteristics and receptor statuses, breast cancer is subdivided into different subtypes. Luminal A and B subtypes are ER positive, HER2 positive tumors are an HER2 enriched subtype, and triple negative breast cancer (TNBC) is another subtype that is devoid of ER, PR, and HER2. The molecular characteristics that differentiate these breast cancer subtypes are a result of genetic and epigenetic alterations. Epigenetic changes are reversible genetic modifications that are responsible in altering the expression levels of specific genes involved in cancer cell proliferation and metastasis. Common epigenetic variations are promoter methylation and histone modifications. DNA methyltransferases (DNMT) is involved in DNA promoter methylation, whereas histone acetylation is activated by histone acetyltransferases (HAT), and histone deacetylases (HDAC) is involved in the removal of acetyl residue from histone. Histone methyltransferases (HMT) and histone demethylases (HDM) are involved in histone methylation. Higher levels of promoter methylation and chromatin modifications of certain genes are the early event of breast cancer development [46,47]. A recent study indicated early epigenetic changes in healthy tissue located adjacent to cancerous breast tissue. These changes were specifically observed on the members of Polycomb Repressive Complex 2 (PRC2) [47]. This study found that initial changes in DNA promoter methylation in pre-neoplastic normal cells indicate that these cells will acquire the properties of cancer cells in the future. Hence, all these studies shed light on the assumption that these epigenetic changes induce the progression of cancer progenitor cells, which later leads to uncontrolled cancer cell growth and metastasis [48]. In breast cancer tissue, heavy promoter methylation is detected for various tumor suppressor genes such as APC, BRCA1, CCND2, CDH1, ESR1, GSTP1, HIN1, P16, RARβ, RASSF1, SFRP1, and TWIST, but not in normal breast tissue [49]. The aggressiveness of breast cancer is associated with high promoter methylation of TWIST, SFRP1, ESR1, P16, and APC in lymph node positive breast cancer cases [49]. While BRCA1 mutations occur in the majority of hereditary breast cancers, BRCA1 gene promoter methylation is associated with sporadic breast cancers [50]. Since epigenetic modifications are reversible, targeting these tumor suppressor genes is a unique approach to prevent cancer incidence and growth.

### 3.1. Epigenetic Modifiers in Clinical Use 

Among epigenetic modifiers, DNMT and HDAC inhibitors are currently available to treat different malignancies. FDA approved DNMT inhibitors 5-azacytidine (Vidaza) and decitabine (5-aza-2’-deoxycytidine) is used to treat myelodysplastic syndrome (MDS) and acute myeloid leukemia (AML). While these DNMT inhibitors have been shown to be effective, higher cytotoxicity, lower drug bioavailability, and drug stability are some of the limitations for these therapies [51]. FDA has also approved four HDAC inhibitors: vorinostat and romidepsin for cutaneous T-cell lymphoma, belinostat for relapsed or refractory peripheral T-cell lymphoma, panobinostat in combination with bortezomib (velcade), and dexamethasone for multiple myeloma. To date, the epigenetic modifiers have received clinical approval to treat blood cancers only. Clinical studies suggested some unwanted effect of these epigenetic modifiers in solid tumors [52]. HDAC inhibitors have been known to induce apoptosis in cancer cells, however some studies demonstrate that certain classes of HDAC inhibitors induce angiogenesis by regulating different signaling molecules [53]. Gatla et al. reported that HDAC inhibitors enhance the expression level of proinflammatory cytokines IL8 and CXCL8 via the activation of IkB kinase, which resulted in enhanced solid tumor growth [54]. Available studies revealed that HDAC inhibitors increase the prosurvival and proinflammatory cytokines mediated through NFkB signaling in solid tumors [55]. The therapeutic usage of approved HDAC inhibitors in solid tumors as a single agent may not be successful.

### 3.2. Epigallocatechin Regulates Tumor Suppressor Genes by Promoter Demethylation

In comparison to Western populations, green tea consumption is high among Asian populations. Current reports are conflicting in regards to the correlation between breast cancer occurrence and green tea consumption. Some reports have shown an inverse correlation, while other reports have identified no benefit of tea consumption on breast cancer prevention or occurrence [56,57]. However, breast cancer risk has been reported to be considerably lower among Asian women compared to Western populations. Green tea is rich in the flavonoid catechin, specifically epigallocatechin-3-gallate (EGCG). Table 1 indicates the amount of EGCG that is present in different dietary sources, with brewed green tea containing the highest amount of EGCG. EGCG exhibits anti-oxidant, anti-inflammatory, and anti-viral activities. It also has characteristic anti-cancer properties such as increased apoptosis and cell cycle arrest. The principal mechanism behind cancer prevention and reduction in tumor growth by EGCG has yet to be elucidated. Nevertheless, compared to other flavonoids, there is more data available on the action of EGCG as an epigenetic modulator in breast cancer. Current studies suggest that EGCG regulates DNA methylation and chromatin remodeling and alters the expression levels of different genes that regulate breast cancer proliferation and metastasis [58,59,60]. Following in vitro and in vivo studies, it was postulated that EGCG inhibits breast cancer proliferation and migration by regulating different tumor suppressor genes via its action of inhibiting DNA methyltransferases (DNMT) and histone deacetylases (HDAC) enzymes [61,62,63,64,65,66].

An early study found that EGCG-treated MCF-7 cells showed a reduction in human telomerase reverse transcriptase (hTERT) promoter methylation and histone H3 Lys9 acetylation [58]. EGCG treatment was also found to enhance hTERT repressor E2F-1 binding at the promoter area. The same research group later found that treatment of both EGCG and a pro-drug of EGCG inhibits both DNMTs and histone acyltransferases (HAT) enzyme activity. This resulted in the downregulation of hTERT expression in ER-positive (MCF-7) and ER-negative (MDA-MB-231) cells [59]. Another study demonstrated that MCF-7 and MDA-MB-231 breast cancer cells were epigenetically modified after treatment with EGCG, genistein, withaferin A, curcumin, resveratrol, and guggulsterone [60]. Withaferin A is a steroidal lactone that is present in the medicinal plant *Withania somnifera* which exhibits anti-cancer activity in breast cancer [68], Guggulsterone is a phytosterol that induces apoptosis in cancer cells [69], and curcumin, a polyphenol, has been known for its anti-cancer and anti-inflammatory effects. The study revealed that EGCG and genistein were more efficient in demethylating different tumor suppressor gene(s) promoter(s) compared to withaferin A, curcumin, resveratrol, and guggulsterone. All of these drug treatments significantly decreased the expression levels of methyl CpG binding protein 2 (MeCP2) in both cell lines. The study ultimately suggested that EGCG, genistein, curcumin, resveratrol, withaferin A, and guggulsterone exhibit cancer preventative actions by epigenetic modulation of tumor suppressor genes [60]. 

Signal peptide-CUB-EGF domain-containing protein 2 (SCUBE2) is a breast cancer tumor suppressor gene that is silenced due to promoter methylation [61]. Overexpression of SCUBE2 reversed epithelial mesenchymal transition (EMT) and associated cellular signaling in breast cancer. A recent study by Sheng et al. reported that EGCG significantly inhibited DNMT activity and demethylated SCUBE2 promoter methylation [62]. This resulted in higher expression levels of SCUBE2 and reduced breast cancer progression.

Different studies have reported on the combination effect of EGCG with other natural products or epigenetic modifiers in breast cancer. Li et al. used two different types of breast cancer cell models to study the effect of the combination of EGCG and sulforaphane at different stages of the disease [63]. The normal human mammary epithelial cells (HMECs) were transfected with SV40 and hTERT to develop precancerous breast cancer cells and a completely transformed breast cancer cell model was developed by the additional transfection of the H-Ras oncogene. This study suggested that a combination of EGCG and sulforaphane treatment induced apoptosis, inhibited cell proliferation, and inhibited enzymatic activities of both DNMT1 and HDAC1 in completely transformed cells compared to pre-transformed cells. Further, this study demonstrated that EGCG and sulforaphane treatment-induced hypermethylation of several key genes plays a significant role in breast cancer tumor progression. It also discovered that tumor growth of breast cancer xenografts was inhibited more by combination treatment with EGCG and sulforaphane than by individual treatment with either of these compounds.

Lewis et al. treated TNBC cell lines (MDA-MB-157 and MDA-MB-231) and ER-positive cell lines (MCF-7) with the combination of suberoylanilide hydroxamic acid (SAHA), HDAC inhibitor, and EGCG [64]. SAHA and EGCG exposure significantly decreased DNMT activity in both TNBC cell lines and the ERα-positive cell line. Furthermore, the combination treatment of SAHA and EGCG not only significantly decreased the oncogenic miR-221/222 expression levels, but also increased the expression levels of p27 and ERα tumor suppressor genes. The histone acetylation on p27 and PTEN promoter was significantly increased with the combination treatment. Triple-negative and ER-negative breast cancers are more aggressive compared to ER-positive breast cancers. A combination of EGCG and the HDAC inhibitor trichostatin-A significantly induced ERα due to increased ERα promoter histone acetylation and dimethylation of H3 lysine 4 (H3K4), but it decreased trimethylation of H3K9 [65]. Furthermore, this study suggested that EGCG and trichostatin-A treatment decreased the binding of the transcription repressor complex Rb/p130-E2F4/5-HDAC1-DNMT1-SUV39H1 to ERα promoter. Another study by Li et al. demonstrated that the combined treatment of EGCG and sulforaphane inhibited DNMT and HDAC1 activity, leading to enhanced ERα expression levels in MDA-MB-231 and MDA-MB-157 cells [66]. An interesting observation of this study was that the treatment of EGCG and sulforaphane decreased ER-negative mammary tumor formation and induced the sensitivity towards tamoxifen therapy [66]. 

Recent research reports have indicated that the anti-cancer action of EGCG occurs through epigenetic modification in different types of breast cancer. Moreover, the combination of EGCG with different dietary constituents or epigenetic modifiers has revealed the influence of other dietary sources on anti-cancer/cancer prevention of breast cancer. The inconsistency observed on the anti-cancer action of EGCG among various clinical and epidemiological studies may be due to the disparity among the different types of breast cancers and variations in dietary intake among the study population.

Most of the above-mentioned *in vitro* studies used various concentrations of EGCG from 5–50 µM/mL concentration range to obtain the anti-cancer activity in different breast cancer cells. Based on the available data, it is difficult to propose the association between the amount of green tea intake and the prevention or therapeutic effect of breast cancer via the modification of epigenetic machinery. An animal study demonstrated that oral treatment of green tea extract exhibited a maximum EGCG plasma concentration of 65 ng/mL [70]. A human clinical study identified that the concentration of EGCG in the blood reached up to a maximum of 0.6 μM after the consumption of 2 to 3 cups of green tea [71]. Furthermore, it has been shown that 0.2–2% of the EGCG was detected in the plasma after 90 min of intake [72]. Another clinical study suggested that the plasma concentration of EGCG can reach up to 326 ng/mL after 1 to 2 h of decaffeinated green tea (maximum up to 4.5 g of solids dissolved in 500 mL of water) ingestion [73]. Further clinical studies are essential to identify the amount of green tea consumption required to initiate epigenetic modification.

### 3.3. Genistein and Daidzein Modulates Histone Modifications 

Isoflavones are the major flavonoids that are present in soybeans. They exist as glycosides, including genistin, daidzin, and glycitin, and can act as both estrogen receptor activators and inhibitors. By the action of β-glucosidases in the small intestine, the glycosides are converted to aglycones (genistein, daidzein, and glycitein) and the gut microbiota largely influence the metabolism and biological action of isoflavones. Microorganisms present in the colon convert daidzein to equol, which is known to have a higher ER binding activity and has also been associated with decreased risk of breast cancer [74]. Studies have suggested that Asian populations are high equol producers compared to Western populations [75]. 

Genistein is an isoflavone that is primarily present in soybeans and soy protein. Table 2 indicates the concentrations of genistein and daidzein that is present in various soy foods. Aqueous washed soy protein has a genistein content that is approximately 10 times higher compared to alcohol washed soy protein. Genistein is also known as phytoestrogen because its structure is very similar to human estrogen and it can bind to ERs with more affinity towards the beta ER compared to the alpha ER. Genistein exhibits both estrogenic and anti-estrogenic activity and thus, genistein shows both anti-cancer and cancer-promoting action [76]. The consumption of soy products is also high among Asian populations and many reports have shown genistein’s preventative action against breast cancer for those who consume a soy-rich diet from an early age [77,78,79]. 

However, other clinical trials have not successfully established the cancer prevention or relapse action of genistein in the Western female population [81]. Various in vitro studies indicated that ER status plays a significant role in the anti-cancer activity of genistein in breast cancer. For example, genistein did not show significant cancer-reducing activity in ER-positive breast cancer cell lines or animal models [78]. 

Li et al. reported that genistein reactivated ERα expression in the MDA-MB-231 cell line and the combination of genistein and trichostatin A synergistically enhanced the ERα expression by enhancing three histone acetylation chromatin markers, acetyl-H3, acetyl-H3K9, and acetyl-H4 and decreasing DNMT1 expression [82].

BRCA1 and BRCA2 genes are mutated or epigenetically silenced in ER-negative and TNBC. Bosviel et al. demonstrated that genistein and daidzein reduce BRCA1 and BRCA2 promoter methylation, which leads to enhanced protein levels of BRCA1 and BRCA2 in breast cancer cell lines [83]. Genistein showed higher DNA demethylating action compared to daidzein. This study also found that genistein and daidzein treatment decreased the expression levels of MeCP2 expression, which is correlated with the whole genome methylation [83]. Another study also observed the same effect when they compared the activity of genistein and EGCG on BRCA1 and ERα promoter methylation [84]. This study concluded that genistein significantly decreased CpG methylation of both BRCA1 and ESR-1 promoter, which leads to increased expression levels of these genes in ER-negative breast cancer. 

Another recent study correlated genistein action on aryl hydrocarbon receptor (AhR) and BRCA1 methylation in ER-negative breast cancer [85]. AhR is highly expressed in TNBC and earlier studies reveal that its activation is positively associated with the epigenetic silencing of BRCA1 [86]. Since BRCA1 is highly expressed in ER-positive breast cancer cell lines (MCF-7), the addition of AhR agonist decreased BRCA1 expression in association with increased promoter methylation [83]. This study experimented with the effect of a genistein diet on different growth stages of transgenic breast cancer mice, such as gestation, lactation, and after weaning, and found that genistein treatment significantly decreased BRCA1 methylation in the mammary glands of female mice offspring compared to control. The researchers demonstrated that the genistein diet inhibited AhR action by inducing BRCA1 promoter methylation. Further, they found that genistein dependent activation of BRCA1 enhanced the ERα expression and allowed sensitization of the anti-estrogen therapy tamoxifen to ER-negative breast cancer or TNBC.

Results of another study in a breast cancer animal model suggested that genistein treatment decreased the carcinogenic activity bisphenol A (BPA), a high-volume chemical that is used in the manufacturing of polycarbonate plastics and containers for foods and beverages [87]. The genistein treatment decreased the BPA induced promoter methylation of different tumor suppressor genes in breast cancer [87]. Another study indicated that soy phytoestrogens such as genistein, daidzein, and equol lead to a reduction in trimethylation marks and an increase in acetylating marks of selected genes EZH2, BRCA1, ERα, ERβ, SRC3, and P300 [88]. 

Paul et al. identified anti-cancer action with the combination of genistein and sulforaphane in breast cancer cells [89]. Sulforaphane is enriched in broccoli sprouts and kale and has been shown to have HDAC inhibiting properties. Combined treatment of genistein and sulforaphane (SFN) in breast cancer cell lines inhibited HDAC and histone methyltransferase (HMT) inhibitor action [89]. Further, this study found that the combination of genistein and sulforaphane significantly decreased KLF4 and hTERT protein levels compared to individual treatment. The combination treatment of genistein and sulforaphane also showed a preventive effect in mammary tumor formation in the transgenic mouse models, C(3)1-SV40. These studies indicate that genistein regulates different epigenetically suppressed genes via promoter demethylation and alters chromatin modification of different gene promoters.

The bioavailability of genistein varies with different soy dietary sources. The maximum plasma concentration was observed in soymilk with the range of 3 to 4 µmol/L [90]. Another study analyzed the levels of daidzein and genistein in the plasma of postmenopausal Thai women after the oral administration of soy beverage versus soy extract capsules. This study identified that the maximal plasma daidzein concentration was approximately 96 ng/mL for both soy beverage and soy extract capsules, whereas the maximum plasma genistein concentration was 116 and 261 ng/mL for soy beverage and soy extract capsules, respectively [91]. Another study also reported that when equal amounts of daidzein and genistein were administered, the plasma concentration of genistein and daidzein were 4.54 and 2.94 µg/mL, respectively, which indicates that the bioavailability of genistein is higher compared to daidzein [92]. In most of the in vitro studies, epigenetic alterations were found with 5–20 µM of genistein. Therefore, further clinical studies are required to analyze the dose of the genistein and the amount of soy that is needed to induce the epigenetic modifications.

### 3.4. Resveratrol Upregulates ATP2A3 Expression

Resveratrol (3,5,4’-trihydroxy-trans-stilbene) is a stilbenoid group of natural phenol that occurs in higher amounts in grapes and red wine [93]. The anti-cancer activity of resveratrol has been evaluated in different types of cancer. Available reports indicate that resveratrol inhibits different stages of cancer progression, such as proliferation and metastasis. A recent study found that resveratrol treatment enhanced the ATP2A3 expression significantly by increasing the H3 lysine 27 acetylation on the ATP2A3 gene promoter and decreased the activity of DNMT and the expression levels of methyl-DNA binding proteins MeCP2 and MBD2 [94]. The ATP2A3 gene plays a significant role in intracellular Ca^2+^ management and normal cell death process, and transcriptional down-regulation of ATP2A3 has been identified in various cancers including breast cancer [95]. Furthermore, HDAC inhibitors enhanced the expression levels of ATP2A3 in breast cancer [94]. In another study, resveratrol combined with grape seed proanthocyanidins significantly decreased the DNMT and HDAC activity in breast cancer cell lines [96]. Moreover, this study showed that resveratrol and proanthocyanidins synergistically inhibited the growth of breast cancer cells. As previously mentioned, AhR agonists increased the promoter methylation of BRCA1 promoter in breast cancer [85,86]. The researchers found that maternal resveratrol pretreatment increased the expression of AhR repressor and increased BRCA1 expression in offspring [97]. Thus, their findings indicate that resveratrol prevents breast cancer development by modulating BRCA1 expression via AhR signaling regulation. 

### 3.5. Quercetin Modulates BRCA1 Expression Level

Quercetin is a flavonoid that is specifically categorized under the flavonol subclass. It is present at varying concentration levels in different fruits and vegetables (Table 3). Isoquercetin (quercetin-3-glucoside) is one of the glycoside forms of quercetin that is present in plants [98]. Reports have suggested that quercetin exhibits anti-cancer effects in breast cancer [99,100,101], however the literature on the epigenetic modifying activity of quercetin in breast cancer is limited. Our earlier study demonstrated that quercetin regulates beta-catenin signaling which leads to the inhibition of EMT in TNBC [99]. Meanwhile, a correlation between β-catenin and HAT has been reported in other cancers [100]. Recently, we showed that quercetin enhances epigenetically silenced BRCA1 expression in TNBC [102]. Furthermore, our study revealed that the combined effect of quercetin and curcumin enhanced BRCA1 expression synergistically and the BRCA1 promoter histone H3K9 acetylation was significantly increased with the combined treatment in a TNBC cell line (MDA-MB-231) [10].

The bioavailability of quercetin is poor as one clinical study found that quercetin plasma concentration was around 0.1 μg/L after 2 h administration of 630 mg quercetin/m^2^ [103]. Another study found that 431 nmol/L was the maximum plasma concentration after 6 h of 150 mg quercetin intake [104]. Further, a study demonstrated that the maximum quercetin concentration was 899 μg/L after 28 days of 1000 mg/day quercetin administration [105]. More in vivo studies are necessary to evaluate the plasma concentration of quercetin after the administration of different natural sources of quercetin. It has yet to be determined the amount of quercetin from dietary sources that is needed to modulate epigenetic changes in breast cancer.

## 4. Conclusions and Further Perspectives

The majority of available reports have indicated that flavonoids have some kind of anti-cancer action in breast cancer [28,29,30]. The epidemiological studies on flavonoids and breast cancer have some limitations, including study design, low number of sample sizes, varying doses of flavonoid consumption, and the subtype of breast cancer. Research also indicates that menopausal status may be a significant contributor to the disparity observed for flavonoid action in breast cancer. 

When considering isoflavones, conflicting reports are available on the action of genistein on breast cancer prevention and promotion [32,33,34,106]. There is considerable literature available showing cancer reducing properties of isoflavone and at the same time, one report suggested that genistein did not protect tumor development in a BRCA1 mutated mouse model [106]. The breast cancer prevention effect was more evident in the East Asian compared to the Western population due to the high consumption of soy-related foods [76,77,78,79]. In the Western world, consumption of isoflavones usually comes from genistein/daidzein supplementation and the amount of isoflavone that is available is lower in supplements compared to soy-related foods [38]. The cancer prevention property of isoflavone is not conclusive based on epidemiological studies. Additional studies are needed to evaluate the action of the same amount of isoflavone in different ethnic populations to establish its breast cancer preventive action. 

This current review also analyzed the literature on the action of various flavonoids as epigenetic modifiers for breast cancer. EGCG is a well-studied flavonoid in breast cancer as an epigenetic modifier. Different studies postulated that EGCG acts as an inhibitor for DNMT and HDAC, resulting in the expression of different tumor suppressor genes [58,59,60,61,62,63,64,65,66]. Furthermore, different studies used EGCG together with another epigenetic modifier or a natural product and showed the combined action on regulating the epigenetic enzymes [64,65,66]. Treatment with EGCG and HDAC inhibitors induced re-expression of ERα in TNBC and ER-negative breast cancer. Similarly, the isoflavone genistein also exhibited DNMT inhibitor action and upregulated BRCA1 and ERα expression by decreasing promoter methylation via the inhibition of AhR in TNBC [83]. In addition, resveratrol, a polyphenol, increased ATP2A3 gene expression by decreasing DNMT activity and enhanced histone acetylation [93]. Table 4 displays the summary of various genes regulated by EGCG, genistein, and resveratrol treatment in breast cancer cells. 

**Figure 1:**
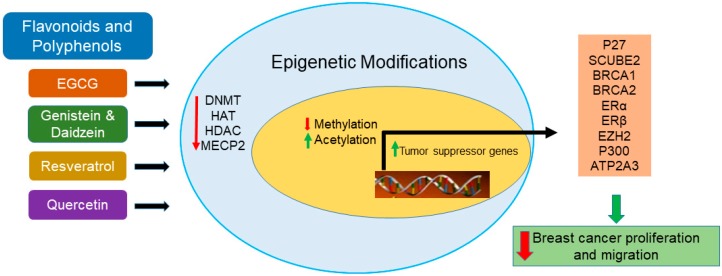
Schematic representation of the action of flavonoids and other polyphenols on epigenetic modifications and induction of tumor suppressor genes.

Available studies indicate that EGCG, genistein, and resveratrol induce tumor suppressor genes via downregulation of DNMT and alteration of chromatin modifications. Furthermore, these studies shed light on the notion that the reduction in breast cancer incidence in Asian women compared to Western populations may be due to the consumption of green tea and soy products. Figure 1 shows a schematic representation of epigenetic modifications of different tumor suppressor genes that are influenced by flavonoids and other polyphenols in breast cancer. The assumption is that the consumption of EGCG, genistein, resveratrol, or quercetin rich dietary sources may protect the healthy tissue that neighbors cancerous tissue from the epigenetic changes. In addition, we assume that the action of flavonoids/polyphenols in regulating these epigenetic machineries may prevent the transformation of normal breast cells to tumor cells. 

Most of the studies related to the association between flavonoids and epigenetic modifications that were included in this review were carried out in different breast cancer cell culture models. Hence, further in-depth in vivo studies are required to evaluate the epigenetic modifying action of these flavonoids in breast cancer. 

## Figures and Tables

**Table 1:** Dietary sources of epigallocatechin-3-gallate.

Dietary Source	mg/100 g Source
Green tea, brewed	70.20
Oolong tea, brewed	34.48
Green tea, brewed (decaffeinated)	26.05
Black tea, brewed	9.36
Pecannut	2.30
Fuji Apple, with skin	1.93
Hazelnut	1.06
Black tea, brewed (decaffeinated)	1.01
Cranberry	0.97
Blackberry	0.68
Raspberry	0.54
Plum	0.48
Red Delicious Apple, without skin	0.46
Pistachio nuts	0.40
Granny Smith Apple, with skin	0.24
Golden Delicious Apple, with skin	0.19
Avocado	0.15
Red Delicious Apple, with skin	0.13
Gala Apple, with skin	0.11
Strawberry	0.11

Bhagwat, S., et al, 2014 [67].

**Table 2:** Dietary sources of genistein and daidzein.

Dietary Source	Serving	Genistein (mg)	Daidzein (mg)
Soybeans, mature seeds (boiled)	½ cup	26.9	26.5
Soy protein concentrate, aqueous washed	3.5 ounces	52.8	38.2
Soy protein concentrate, alcohol washed	3.5 ounces	5.8	5.3
Soybeans, dry roasted	1 ounce	21.2	17.4
Soybeans, green, boiled (edamame)	½ cup	6.3	6.7
Miso	½ cup	32	22.6
Soy milk, low-fat	1 cup	3.7	2.4
Tofu, soft	3 ounces	10.1	8.1
Tofu, yogurt	½ cup	12.3	7.5
Tempeh	3 ounces	30.7	19.3
Tempeh, cooked	3 ounces	18	11.1
Soy burger, unprepared	1 patty	3.5	1.6
Soy sausage	3 links	6.9	3.3
Soy cheese, cheddar	1 ounces	0.6	0.5

“Soy Isoflavones.” Linus Pauling Institute [80].

**Table 3:** Dietary sources of quercetin.

Dietary Source	mg/100 g Source	Dietary Source	mg/100 g Source
Elderberry juice	108.16	Mustard greens, raw	8.80
Radish leaves, raw	70.37	Mizuna (Japanese mustard)	8.55
Chokeberry juice	68.17	Arugula, raw	7.92
Wild rocket, raw	66.19	Blueberry	7.67
Cilantro leaves, raw	52.90	Red leaf lettuce, raw	7.61
Yellow wax hot pepper, raw	50.63	Red swiss chard, raw	7.50
Juniper berry, ripe	46.61	Rowanberry, raw	7.40
Green juniper berry, unripe	42.81	Chokeberry juice	6.49
Red onion, raw	39.21	Fig, raw	5.47
Radicchio, raw	31.51	Crowberry, raw	5.45
Watercress, raw	29.99	Chives, raw	4.77
Hartwort leaves	29.30	Acerola, west Indian cherry	4.74
Ancho pepper	27.60	Blueberry, frozen	4.64
White onion, pan fried	26.90	Cranberry, dried	4.50
Corn poppy leaves	26.30	Kale, canned	4.50
Onion, cooked	24.36	Bayberry, raw	4.36
Hawthorn leaves, raw	24.10	Brussels sprout, cooked	4.33
Nalta jute, raw	23.53	Green leaf lettuce, raw	4.16
Currant	22.85	Red Delicious Apple, with skin	3.86
Okra, raw	20.97	Gala Apple, with skin	3.80
Apple, skin only	19.36	Golden Delicious Apple, with skin	3.69
Wild bog whortleberry, frozen	17.70	Blackberry, raw	3.58
Sour cherry, powder	17.44	Bay leaves	4.33
Sweet potato leaves, raw	16.94	Green leaf lettuce, raw	3.19
Juneberry or Saskatoon berry	16.64	Concord grape	3.11
Cranberry juice	16.41	Bilberry, raw	3.04
Buckwheat	15.38	Yellow snap bean, cooked	3.03
Asparagus, cooked	15.16	Granny Smith Apple, with skin	2.54
Cranberry, raw	14.84	Cranberry sauce, canned	2.40
Asparagus, raw	13.98	Fuji Apple, with skin	2.35
Goji berry (wolfberry), dried	13.60	Broccoli raab, raw	2.25
Lingonberry (cowberry)	13.30	Purple plum, raw	2.19
Spring onion leaves	12.60	Tomato juice, canned	1.19
Black diamond plum, with peel	12.45	Red grape	1.04
Cranberry bush berry, raw	10.73	Watercress, steamed	0.63
Sweet potato leaves, cooked	9.84	Golden Delicious Apple, without skin	0.51
Arctic brambleberry	9.10	Red Delicious Apple, without skin	0.41

Source: Bhagwat, S.; et al 2014 [67].

**Table 4:** Summary of epigenetic modification of different genes.

Flavonoid	Epigenetic Modification	Breast Cancer Cell Line	Regulated Genes	Reference
EGCG	↓ Promotermethylation ↓ H3Lys9 Acetylation	MCF-7	↓ hTERT	Berletch et al., 2008 [58]
EGCG	↓ DNMT ↓ HAT	MCF-7 & MDA-MB-231	↓ hTERT	Meeran et al., 2011 [59]
EGCG	↓ Promoter methylation ↓ DNMT	MDA-MB-231	Signal peptide-CUB-EGF domain-containing protein 2 (SCUBE2)	Lin et al., 2014 [61] Sheng et al., 2019 [62]
EGCG & SAHA	↓ DNMT ↑ Histone Acetylation	MCF-7, MDA-MB-231 & MDA-MB-157	↑ ERα, p27, & E-cadherin	Lewis et al., 2018 [64]
EGCG & Sulforaphane	↓ DNMT& HDAC	HMECs MDA-MB-231 & MDA-MB-157	↑ DCBLD2, ↓ SPET9↑ ERα	Li et al., 2016 [63] Li et al., 2017 [66]
EGCG & Trichostatin A	↑ Histone Acetylation ↑ H3K4Me2 ↓ H3K9	MDA-MB-231	↑ ERα	Liet al., 2010 [65]
Genistein & Trichostatin A	↑ Histone Acetylation ↓ DNMT	MDA-MB-231	↑ ERα	Li et al., 2013 [82]
Genistein and Daidzein	↓ Promoter methylation ↓ MeCP2	MDA-MB-231	↑ BRCA1 & BRCA2	Bosviel et al., 2012 [83]
Genistein	↓ DNMT1	UACC-3199 HCC38	↑ BRCA1 & ERα	Romagnolo et al., 2017 [84] Donovan et al., 2019 [85]
Genistein	↓ H3K27Me3 & H3K9Me3, ↑ H4K8Ac and H3K4Ac	MDA-MB-231	↑ EZH2, BRCA1, ERα, ERβ, SRC3 and P300	Dagdemir et al., 2013 [88]
Genistein and Sulforaphane	↓ HDAC & HMT	MCF-7 & MDA-MB-231	↓ KLF4 and hTERT	Paul et al., 2018 [89]
Resveratrol	↑ H3K27Ac ↓ DNMT, MeCP2 & MBD2	MDA-MB-231	↑ ATP2A3	Izquierdo-Torres et al., 2019 [94]
Resveratrol	↓ DNMT1		↑ BRCA1	Papoutsis et al., 2015 [97]

“↓”, Lower, “↑” Higher.
